# A review of coxiellosis (Q fever) and brucellosis in goats and humans: Implications for disease control in smallholder farming systems in Southeast Asia

**DOI:** 10.1016/j.onehlt.2023.100568

**Published:** 2023-05-22

**Authors:** Rebekah J.L. Burns, Kim Khanh Le, Jarunee Siengsanun-Lamont, Stuart D. Blacksell

**Affiliations:** a Mahidol-Oxford Tropical Medicine Research Unit, Faculty of Tropical Medicine, Mahidol University, Bangkok, Thailand; b Lao-Oxford-Mahosot Hospital-Wellcome Trust Research Unit (LOMWRU), Mahosot Hospital, Vientiane, Lao Democratic People’s Republic; c Centre for Tropical Medicine & Global Health, Nuffield Department of Medicine, University of Oxford, Oxford, United Kingdom

**Keywords:** Q fever, Coxiellosis, Brucellosis, Asia, Small ruminants

## Abstract

*Coxiella burnetii* and *Brucella* spp. are pathogenic bacteria that can cause large-scale outbreaks in livestock. Furthermore, these infectious agents are capable of causing zoonotic infections and therefore pose a risk to the close relationship between farm households and their livestock, especially goats. A review of seroprevalence studies of *Coxiella burnetii* and *Brucella* spp. in domestic goats demonstrated large differences in the total number of samples tested in different regions and countries. This review aims to provide information on coxiellosis (Q fever in humans) and brucellosis in goats concerning the characteristics of the causative agent, surveillance, and available prevention and control measures at a global level. Implications for *Coxiella burnetii* and *Brucella* spp. infections in domesticated goats in Southeast Asia are discussed.

## 1 Introduction

*Coxiella burnetii* (causative agent of Q fever in humans) and *Brucella melitensis* are important bacterial zoonotic pathogens associated with goats [1], other livestock and humans [2]. These pathogens can cause large-scale outbreaks due to their low infectious dose, environmental resistance and ability of airborne spread via aerosolisation of the pathogens [3]. Furthermore, *C. burnetii* and *Brucella* spp. can economically impact rural livelihoods by reducing productivity due to reproductive loss in livestock herds [4,5].

Q fever and brucellosis are challenging to diagnose and treat in humans due to their non-specific clinical signs and intracellular nature [3,4]. They are considered a higher risk for those working with livestock and wildlife through occupational exposure. Both pathogens are potential biothreats classified as “Select Agents” in the USA [6,7] and have long been associated as major causes of laboratory-acquired infections and unintended laboratory releases [[8], [9], [10], [11], [12]].

This review presents characteristics of *C. burnetii* and *Brucella* spp., preventive and therapeutic measures. This article aims to provide updated information on coxiellosis and brucellosis in goats, including the detailed characteristics of the causative agent, available prevention and control measures, and the seroprevalence of infection worldwide while focusing on its relevance to Southeast Asia.

### 1.1 Smallholder goat farming in Southeast Asia

In Southeast Asia, smallholder goat farming typically entails raising a small number of goats, fewer than 20, on a small plot of land. These goats are usually raised for their meat and milk and are an essential source of protein and income for numerous rural families in the region [13,14]. Goats are frequently raised in free-range systems in Southeast Asia, where they can graze on natural pasture or browse on shrubs and trees. They may also be fed crop residues, grasses, and other locally accessible feeds [[15], [16], [17]]. Goats are browsers requiring minimal inputs and can be grown on marginal land, making them a viable option for small-scale farmers with limited resources.

Additionally, goats are relatively easy to handle and manage, making them a good option for farmers with limited experience with livestock. Lack of market access and veterinary services is among the most significant obstacles facing smallholder goat farmers in Southeast Asia and elsewhere [18]. However, with booming economies throughout the region, there is an increased demand for goat meat in Vietnam and China [19]. In addition, limited access to veterinary services may make managing disease outbreaks difficult and preventing goat herd health issues complex. Many goat diseases are zoonoses, such as *C. burnetii* and *Brucella melitensis* [19,20], which could infect the smallholder and their families. Peste des petits ruminants (PPR) [21] also remains a threat throughout the region. Despite these obstacles, goat farming remains an important source of income for many rural households in Southeast Asia.

## 2 Coxiella burnetii

### 2.1 Introduction and history

Q fever in humans is caused by the agent *Coxiella burnetii* of the *Legionellales* order. Q “Query” fever was first described in 1937 following an outbreak of febrile illness in Queensland abattoir workers [22]. There have been several outbreaks of Q fever in humans, often involving goats. The largest outbreak in the Netherlands (2007–09) had 3523 human cases recorded two years following a kidding season by recently imported goats [23,24]. Q fever has been reported worldwide except in New Zealand [20,25].

### 2.2 The infectious agent

*C. burnetii* is an obligate intracellular gram-negative coccobacillus responsible for Q fever in humans and coxiellosis in ruminants. It can infect many animals, including ruminants, dogs, cats, and birds, and can potentially be shed in marsupial faeces [26,27]. The primary spread of the pathogen between mammals is via aerosols. Ticks also play an epidemiological role, as *C. burnetii* can replicate and be shed in tick faeces [28,29]. *C. burnetii* has been classified as a Category B biological warfare agent by the US Centers for Disease Control and Prevention [6] due to the agent's extremely high infectivity, ability to cause disease, ability to withstand harsh environmental conditions consistently and can be produced universally on a mass scale [6].

### 2.3 Transmission and spread

The primary risk for transmission and spread of *C. burnetii* is during ruminant parturition [26]. *C. burnetii* has an affinity for placental tissues, particularly the trophoblast cells of the allantochorion [30]. Infection in livestock is largely asymptomatic: however, it can cause abortion, weak newborns, and decreased milk production [30]. Therefore, in affected animals exhibiting normal parturitions, *C. burnetii* can be aerosolised, generating a major source of exposure to other mammals, including humans [30]. *C. burnetii* has been found in blood, lungs, spleen and liver in the acute phase following inoculation of small ruminants hence preparing meat for food in smallholder situations is a significant risk [30]. Further, *C. burnetii* has been isolated from the milk of small and large ruminants, making consuming unpasteurised milk products an infection risk [30].

### 2.4 Human Q fever

Q fever in humans is likely underdiagnosed due to its nonspecific symptoms and the lack of clinician awareness [31]. During the early stages of Q fever, most patients are asymptomatic or have flu-like symptoms, including fever, fatigue, and muscle pain [26,32]. Human populations with higher seroprevalence of *C. burnetii* tend to be situated in rural areas, with animals also having a high seroprevalence. In Cyprus, Pakistan and the Netherlands, a high seroprevalence in humans was correlated to areas with high seropositivity of goats and reported goat abortions [[33], [34], [35]]. A recent human study on the Queensland (Australia) population indicated similar Q fever seroprevalence rates in metropolitan and rural populations [36], highlighting that Q fever can also affect those in low-risk groups. Human-to-human transmission of Q fever is extremely rare [25].

### 2.5 Prevention

Vaccines are available to prevent Q fever in humans [37,38], although it is not 100% effective, and failures have been recorded [39]. Furthermore, the vaccine is only available in Australia to certain occupations with potential exposure risk [38]. Veterinarians and support staff have a higher potential for Q fever infections via occupational exposure [40,41]. In Australia in 2014, a cross-sectional online study was conducted on two groups, including veterinary doctors (890 responses) and veterinary nurses (852 responses), to ascertain vaccination status and assess knowledge and attitudes regarding Q fever vaccination. According to the findings, veterinarians had higher vaccination rates than veterinary nurses i.e., 74% versus 28%, respectively [31].

### 2.6 Seroprevalence and risk studies in goats worldwide

Goats have been associated with outbreaks of *C. burnetii,* including in the Netherlands (2007–2020) [23,24], China (2018–2019) [42] and Australia (2012–2014) [43]. Exposure rates vary greatly with geographical location and are summarised in Table 1. Interestingly, from 2020 to 2023, 16*C. burnetii* seroprevalence studies that examined goat samples (and other livestock) have been published in peer-reviewed reports (Table 1). Many of these studies were performed in developing countries in Africa (Kenya [44], Mali [45], Namibia [46]), South Asia (India [47], Nepal [48], Pakistan [49]) and Southeast Asia (Cambodia [50], Thailand [51]), where smallholder livestock systems are common. One of the likely reasons for the increased number of seroprevalence studies is the availability of accurate screening tests, such as the ELISA [52], for detecting *C. burnetii* antibodies in a range of species. The studies have revealed significant seroprevalence levels in cross-sectional studies ranging from 0.8% in Nandi County in Kenya [44] to 74.7% in Marsabit County in northern Kenya [53]. Many of the studies were multispecies surveys, including cattle and sheep, in the analysis to better understand the risk factors associated with *C. burnetii* infection dynamics in smallholder communities.

**Table 1:** *C. burnetii* seroprevalence studies in goats worldwide by country and region.

Country	Region⁎	Area⁎	Seropositivity (%)	Overall number of samples tested (n)	Year Published	Author
Kenya	Africa	Sub-Saharan Africa	0.8%	132	2022	Kiptanui et al. [44]
Kenya	Africa	Sub-Saharan Africa	74.7%	1876	2022	Muema et al. [53]
Kenya	Africa	Sub-Saharan Africa	15.2%	1333	2022	Mwololo et al. [100]
Namibia	Africa	Sub-Saharan Africa	2.9%	52	2022	Samkange et al. [46]
South Africa	Africa	Sub-Saharan Africa	18.4%	216	2023	Magadu et al. [103]
Mali	Africa	Sub-Saharan Africa	16.9%	290	2022	Dione et al. [45]
China	Asia	Eastern Asia	22%	150	2016	El-Mahallaway et al. [58]
China	Asia	Eastern Asia	4.8%	1157	2018	Li et al. [93]
Cambodia	Asia	South-eastern Asia	7.2%	540	2022	Siengsanan-Lamont et al. [50]
Laos	Asia	South-eastern Asia	4.1%	1458	2018	Burns et al. [19,101]
Thailand	Asia	South-eastern Asia	3.5%	516	2017	Doung-ngerm et al. [59]
Thailand	Asia	South-eastern Asia	69%⁎⁎	118	2022	Rerkyusuke et al. [51]
Iran	Asia	Southern Asia	65.8%	76	2009	Khalili et al. [98]
Iran	Asia	Southern Asia	45.5%	224	2021	Fakour et al. [99]
India	Asia	Southern Asia	1.6–11.5%	411	2020	Leahy et al. [47]
Nepal	Asia	Southern Asia	4.4–23.2%	242	2021	Paudyai et al. [48]
Pakistan	Asia	Southern Asia	17.1%	158	2022	Amin et al. [49]
Pakistan	Asia	Southern Asia	33.2%	271	2016	Zahid et al. [33]
Cyprus	Asia	Western Asia	48.2%	420	2006	Psaroulaki et al. [35]
United Arab Emirates	Asia	Western Asia	32.1%	449	2022	Barigye et al. [105]
Hungary	Europe	Eastern Europe	31.0%	71	2021	Dobos et al. [97]
Estonia	Europe	Northern Europe	0%	18 (flocks)	2023	Neare et al. [95]
Republic of Ireland	Europe	Northern Europe	0.3%	590	2011	Ryan et al. [102]
Croatia	Europe	Southern Europe	100%⁎⁎	16	2022	Tomljenovic et al. [94]
Greece	Europe	Southern Europe	6.5%	61	2009	Pape et al. [96]
Spain	Europe	Southern Europe	8.7%	115	2010	Ruiz-Fons et al. [54]
Netherlands	Europe	Western Europe	21.4%	2828	2011	Schimmer et al. [34]
Switzerland	Europe	Western Europe	3.4%	321	2015	Magouras et al. [104]
Australia	Oceania	Australia and NZ	51.5%	3720	1981	Hein et al. [43]

⁎ Based on United Nations designations (https://unstats.un.org/unsd/methodology/m49/).

⁎⁎ Symptomatic cases, not cross-sectional serology.

Various risk factors for seropositive animals have arisen from these studies; in Spain, older animals were at higher risk [54]; in Pakistan and Australia, female animals were at higher risk [33,43]; in Pakistan, goats infested with ticks, history of abortion, retention of foetal membranes, single breeds in herds, and feed mismanagement were all risk factors [33]. In the Netherlands, proximity to other positive farms, dogs or cats in the goat stable, large farms with >800 animals and artificial insemination were all significant risk factors [34].

### 2.7 Coxiella studies in Southeast Asia

A few available studies describe the prevalence of *C. burnetii* antibodies in Southeast Asia; however, many focus on integrated livestock systems rather than smallholder goat raising.

In Lao PDR, initial investigations of *C. burnetii* seroprevalence revealed that between 3 and 4% of cattle and buffalos were seropositive [55] with the province of Xayaboury, bordered by Thailand, having increased prevalence [55,56]. Subsequently, studies exclusively on goat serum from five provinces (Vientiane Capital, Xayaboury, Xiengkhuang, Savannakhet and Attapeu) found overall individual *C. burnetii* seropositivity was 4.1% significant risk factors for seropositivity included where are the animals were located by province (Vientiane Capital, *p* = 0.05), breed (introduced Boer mixed breed, *p* = 0.006) and age (goats ≥3 years old, *p* = 0.014) [19].

In Cambodia, a recent seroprevalence study in six provinces [57] concluded that the overall true prevalence of *C. burnetii* antibodies in 540 goats was 7.2%. The two risk factors for *C. burnetii* seropositivity were sex (*p*-value = 0.0005) and location of the commune (p-value <0.0001), with the odds ratio of *C. burnetii* seropositive female goat being significant at 9.7 (95% CI 2.7, 35.5) times higher than male. Another seroprevalence study conducted in China indicated that 22% of goats (29% of cattle and 3% of pigs) were *C. burnetii* seropositive [58] and also found that 25% of humans were *C. burnetii* seropositive and associated with increasing age [58].

In Thailand, [59], animal sera from the provinces of Chiangmai and Nakornratchasima were tested, and 64 (3.9%) of the 1632 tested were seropositive with goats (3.5%; 18/516) and sheep (2.1%; 1/48) (dairy cattle (4.6%; 45/988), and PCR detected four positive milk samples out of sixty (6.7%). In the same study, 12.6% of 661 human samples were positive by ELISA [59]. Other studies in northern Thai provinces have reported similar seroprevalence results in cattle (5.2% [60], 4.6% [59], 6% [61]) and goat (3.5%) [60] samples. The first confirmed human cases of Q fever in Thailand were reported in 2003 [62], where 1.3% of fever presentations (*n* = 9 cases; *n* = 678 total) in north-eastern Thailand were confirmed- positive. All cases were rice farmers, and their animals were chicken and cattle [62]. Q fever endocarditis fatalities were reported in two male cattle farmers in northern Thai provinces [63].

## 3 *Brucella* spp

### 3.1 Introduction and history

Brucellosis is an infectious bacterial disease caused by species of the genus *Brucella.* Brucellosis can be documented as far back as 1600 BCE in Egypt and has continued to cause febrile illness globally. Brucellosis, also known as “Malta Fever”, is named after the febrile illness common among British soldiers stationed in Malta during the 19th century [3].

### 3.2 The infectious agent

Bacteria of the *Brucella* genus are gram-negative coccobacilli of the Proteobacteria phylum. Within the *Brucella* genus are nine species, of which seven affect terrestrial animals; *B. abortus, B. melitensis, B suis, B. ovis, B. canis, B. neotomae* and *B. microti* [2]. Within species are biovars: *B. melitensis* has three biovars which differ in geographical location rather than pathogenicity.

*Brucella* spp. is a facultative intracellular pathogen of mammalian hosts that survives and multiplies within phagocytic cells [2,64]. *Brucella* spp. can enter the host across mucosal membranes [3]. The O-side-chain on the LPS appears involved in invasion mechanisms and protection from oxidative killing, cationic peptides and complement-mediated lysis [65]. *Brucella* spp. avoid destruction by resisting fusion with lysosomes [66]. *Brucella* spp. organisms ultimately become sequestered within phagocytes of the reticuloendothelial system (RES), such as lymph nodes, liver, spleen and bone marrow, where they multiply in modified phagosomes termed “brucellosomes” [2]. Dissemination to other organs occurs via lymph and blood [67]. A specific tropism exists for reproductive tissues, including the placenta, mammary lymph nodes and ducts, and testicular tissue [1].

*Brucella* spp. are classified by the US Centers for Disease Control and Prevention as “bioterrorism agents” due to their stability, ability to cause mass disease with a low infectious dose, ability to be aerosolised and potential for mass dissemination of the pathogen [3,64,68]. *Brucella* spp. can survive long periods in the environment, surviving freezing and thawing and can live for up to 4 months in milk, urine, water, faeces and damp soil [[1], [2], [3]]. Cooler conditions favour the survival of *Brucella* spp. [2]. Brucellosis is a systemic infection that can involve any organ or tissue of the body [2]. Common routes of infection include direct inoculation through cuts and abrasions in the skin, inoculation via the conjunctival sac of the eyes, inhalation of infectious aerosols, and ingestion of infectious milk or milk products [2,3]. The latter is an unlikely aetiology in Southeast Asia as it is the lowest milk-drinking region in the world [69]. The primary source of brucellosis infection in humans is infected birth fluids from animals [3].

### 3.3 Global incidence

Globally, the seroprevalence of *Brucella* spp. in goats varies from region to region and within countries Table 2. It is difficult to compare studies due to differences in serology methodologies, sampling and analysis. *B. melitensis*-free status has been given to Sweden, Denmark, Finland, Germany, the UK, Austria, Netherlands, Belgium, France, Luxembourg, Australia and New Zealand [70]. Generally, a higher human incidence of brucellosis occurs where there is a higher *Brucella* spp. seroprevalence. The true incidence of brucellosis in developing countries may be higher as inadequate medical facilities may lead to underdiagnosis.

**Table 2:** Seroprevalence studies of *Brucella* spp. in goats worldwide by country and region.

Country	Region⁎	Area⁎	Seropositivity (%)	Overall number samples tested	Year Published	Author
Egypt	Africa	Northern Africa	8.6%	360	2022	Fereig et al. [106]
Libya	Africa	Northern Africa	31%	340	2010	Ahmed et al. [79]
Namibia	Africa	Sub-Saharan Africa	23.0%	52	2022	Samkange et al. [46]
Nigeria	Africa	Sub-Saharan Africa	2.8%	2827	2014	Ogugua et al. [76]
Uganda	Africa	Sub-Saharan Africa	10.0%	1446	2001	Kabagambe et al. [73]
Mexico	Americas	Latin America and the Caribbean	9.3%	12,127	2016	Marin et al. [75]
Tajikistan	Asia	Central Asia	5.0%	407	2016	Rajala et al. [78]
Cambodia	Asia	South-eastern Asia	0.4%	540	2022	Siengsanan-Lamont et al. [50]
Laos	Asia	South-eastern Asia	1.4%	1458	2018	Burns et al. [19]
Malaysia	Asia	South-eastern Asia	0.9%	119,799	2015	Bamaiyi et al. [77]
Thailand	Asia	South-eastern Asia	1.4%	94,722	2016	Sagarasaeranee et al. [74]
Thailand	Asia	South-eastern Asia	46.6%⁎⁎	118	2022	Rerkyusuke et al. [51]
India	Asia	Southern Asia	9.9–22.0%	411	2020	Leahy et al. [47]
Iran	Asia	Southern Asia	13.9%	360	2014	Ebrahimi et al. [107]
Türkiye	Asia	Western Asia	26.9%	104	2016	Bora et al. [108]
Portugal	Europe	Southern Europe	0.4%	51,298	2013	Coelho et al. [72]
Spain	Europe	Southern Europe	0.1%	21 herds	2000	Reviriego et al. [71]

⁎ Based on United Nations designations (https://unstats.un.org/unsd/methodology/m49/).

⁎⁎ Symptomatic cases, not cross-sectional serology.

Several risk factors arise from these serological studies, including exposure to infected animals and trading of animals. Contact with other flocks and free-range grazing has been described as a risk factor for seroprevalence in goat herds [[71], [72], [73], [74]]. This could explain the general trend of widespread brucellosis occurring in small ruminants in developing nations. These countries tend to have smallholder pastoralists who are either landless or land-poor and move their goats about or allow them to free-range with other goats during the day [67]. High herd numbers and subsequent herd density risk brucellosis as close contact with other animals may make the spreading of the disease more accessible [72,75]. Areas with increased international trade are also at higher risk for expanded seroprevalence [76,77]. In these studies, authors noted that the disease could be introduced from other regions, or trade induces stress on animals and increases herd density, allowing the pathogen to spread [76,77]. “Mis-management” [72] and lack of veterinary care [73] were noted as risk factors. Generally speaking, “mismanagement” was defined as allowing goats to have frequent contact with their manure and lack of appropriate feed [72]. Older animals tend to have higher seroprevalence, probably due to higher exposure to the pathogen [55,56,78]. In some studies, females have higher seroprevalence than males, possibly due to farmers breeding females for longer and having more opportunities for exposure to the pathogen [79].

Breed and genetics may affect seropositivity. In Mexico, native goat breeds adapted to harsh desert conditions were less likely to be seropositive than imported dairy breeds [75]. In Bangladesh and Malaysia, exotic breeds were significantly more likely to have *Brucella* spp. seropositivity than native breeds [80,81]. Generally, dairy goat breeds are more susceptible to brucellosis than meat breeds as contact with contaminated milk and most reproductive products (i.e., placenta, discharge, amniotic fluid*.*) allows for more opportunities for goats to be challenged by the pathogen [2]. The *Nramp1* gene is critical in innate immunity by enhancing macrophage ability to kill bacteria and positively influences adaptive immunity. Cattle with the *Nramp1* gene are correlated with a natural resistance against brucellosis [82,83]. Further, studies of Nelore cattle breeds, which have been adapted to harsh climates, have macrophages that are more effective in controlling intracellular replication of *B. abortus* than Holstein macrophages, suggesting a higher degree of natural resistance [84].

### 3.4 Brucellosis studies in Southeast Asia and other parts of Asia

Despite limited peer-review articles published regarding brucellosis in South East Asia, evidence suggests that the pathogen is endemic. Five provinces in Lao PDR (Vientiane Capital, Xayaboury, Xiengkhuang, Savannakhet and Attapeu) tested 1458 goat serum samples for Brucella seropositivity resulting in an overall prevalence of 1.4%. They indicated that province (Vientiane Capital, *p* < 0.001), breed (introduced Boer mixed breed, p < 0.001), production system (commercial, p < 0.001), age (adult, *p* = 0.004), and farm size (large, 0.001) were all significant risk factors seropositivity for *Brucella* spp. [19]. Previous studies on cattle in Laos have reported that *Brucella* spp. is not widespread, with only 0.2% of cattle in 2012 and 0.3% of cattle in 2017 being seropositive to *Brucella* spp. [55,56].

In Thailand, the first case of brucellosis was reported in 1970 in a 34-year-old male farmer, and the second case was documented in 2004 [85]. In 2014, there were five cases of brucellosis; in one case, in 2015, most of the patients were farmers with risks of exposure to contaminated goat parts and drank goat milk [85]. Following an outbreak of undulant fever in 2006 in a village northeast of Bangkok, it was determined that 43.5% of villagers were *B. melitensis* seropositive with risk factors including recent contact with goats during parturition and eating raw goat meat [86]. The brucellosis situation in Thailand can be attributed to the significant increase in goat farming in the country since 2000 [74]. *Brucella* seroprevalence in Thailand from 2013 to 2015 in goats and sheep was 12.1% (438/3626) at the herd level; individual goat seroprevalence was 1.4% (1297/94,722), and sheep seroprevalence was 1.6% (139/8658) [74]. At the herd level, only free-ranging animals were substantially associated with brucellosis infection in small ruminants [74]. Another recent study examined 118 samples from goats in north-eastern Thailand, including 85 clinical reproductive disorder cases for *C. burnetii*, *Chlamydophila abortus*, and *Brucella* spp. Results showed that 69% (81/118 cases) were seropositive for Q fever (*n* = 55; 46.6%), brucellosis (*n* = 8; 6.8%), and chlamydiosis (*n* = 18; 15.3%). The study highlighted that buck circulation between herds was a risk factor for diseases (Odds ratio = 109.29) [51]. This fact highlights the importance of biosecurity in herd management to prevent the introduction and spread of brucellosis.

In Cambodia, a recent seroprevalence study [57] in six provinces concluded that the overall true prevalence of *Brucella* spp. antibodies in 540 goats was 0.1% (95% CI 0.0, 1.0). Brucellosis was previously detected in Myanmar, where dairy cows had *Brucella* spp. DNA identified the milk via PCR [87]. In Vietnam, a report from Binh Thuan province indicated that 14.8% of persons presenting febrile illness using the Rose Bengal Agglutination test for *Brucella* spp. However, the study suggested that the evidence demonstrated people's exposure to *Brucella* spp., it did not cause significant health effects on them [88].

Brucellosis has been reported in China, the only region to have developed a human vaccine, although it is not efficacious or safe for use [89]. In one study, rural farmers did not demonstrate antibodies to *Brucella* spp., yet 14.5% of abattoir workers in the same village were positive [90]. A survey in Assam and Odisha states in Eastern India tested 411 goats, resulting in 22% and 9.8% Brucella seroprevalence, respectively [47]. None of the farmers interviewed were aware of brucellosis. [47].

### 3.5 Prevention and control

Reduction in human incidence is usually coupled with managing animal disease and food hygiene. Pasteurisation effectively kills *Brucella* spp.*,* and thoroughly cooking meat is advised. Although cultural traditions are difficult to influence, consuming raw placentas, milk and meat is not recommended. To reduce transmission between a herd, parturition material, especially aborted fetuses and placentae, should be removed and destroyed by incineration before other animals, including farm dogs and cats, and children can be exposed to them [1]. Faeces are cleaned up daily. Regular disinfecting more than three times yearly reduces incidence in a herd [71]. Replacement stock should only be purchased from *Brucella*-free herds [2].

Vaccines are available for both *B. abortus* and *B. melitensis* in livestock, and programs have successfully eradicated *Brucella* spp. from regions. However, entirely satisfactory vaccines are not currently available. Vaccine methods are only recommended after other control measures have failed. The Rev. 1 *B. melitensis* vaccine for caprine and ovine brucellosis control is created from killed bacteria strains [91]. When appropriately used, it causes long-lasting protection against natural infections for most animals, reducing the zoonotic spread of the disease [92]. The vaccine can induce abortions in pregnant nannies and cause the shedding of the organism in animal milk [2,91]. Hence, only sexually immature females are often included in vaccination programs. The vaccine must be coupled with husbandry, test, and cull methods because it is not absolute. Test and cull methods are challenging to implement as they require a political and financial commitment to authorities and provide compensation to farmers. The World Organization for Animal Health (WOAH, formerly OIE) does not recommend antibiotic treatment of animals [2].

Antibiotic treatment for acute human brucellosis usually involves tetracyclines such as doxycycline for six weeks, with aminoglycosides in addition to the first three weeks of treatment. This combination is prolonged for chronic brucellosis treatment [2]. Human vaccines are only available in China and were used in the Soviet Union, although they are reportedly not efficacious or safe for use [3,89,90]. However, the human vaccine has been demonstrated to induce human brucellosis and severe allergic reactions and is only “recommended for human use in high-risk populations” [89].

## 4 Conclusion

*C. burnetii* and *Brucella* spp. are important zoonotic diseases across Southeast Asia and elsewhere because they can have significant health and economic effects on humans and livestock. Both diseases can cause illness, disability, and even mortality in humans, while they can reduce livestock productivity and cause reproductive losses.

However, practical considerations for controlling both diseases in goats in settings with limited resources still need to be solved. It is important that global (WHO, WOAH) and regional organisations (ASEAN), including national governments, invest in disease control strategies and increase awareness of *Coxiella* and *Brucella* infections as important zoonotic infections. Strategies typically used can mitigate the spread of these bacteria and reduce the risk of zoonotic infections in humans listed below; however, it is difficult to know how practical many of these measures would be in a low-resource setting.

- Biosecurity: The implementation of biosecurity measures can prevent the introduction and dissemination of *Coxiella* and *Brucella*. This may involve isolating infected animals from healthy animals, avoiding contact with wild animals, and practising good hygiene, such as washing hands and apparatus between animals. This is especially important following parturition for both diseases. The quarantine of new animals into an existing herd is also a simple, practical measure for biosecurity.
- Vaccination: Vaccination is an economical means of preventing the spread of these diseases; however, access to vaccine supplies and administration per the recommended schedule and dosage will be a challenge, depending on the location of the smallholder farmers.

The above-mentioned practical measures focusing on biosecurity and farmer education are likely to have the greatest success; although vaccination remains an option would require government support and infrastructure.

There is an ongoing need for clinical training in the region to include both diseases in the workup for pyrexia of unknown origin and to emphasise to veterinary students and para-veterinarians the zoonotic nature of these pathogens. Additionally, there is a need for improved diagnostic capabilities for Q fever in both animal and human fields [44]. The final additional control measures, 1) testing and culling and 2) treatment with antibiotics, are problematic in a low-resource, developing-country setting due to a lack of compensation for culling, lack of testing and limited access to antibiotic treatment.

To control the spread of both diseases, it is vital to continue human and animal surveillance to understand better the epidemiology of coxiellosis and brucellosis in the South East Asian region and plan and manage control and eradication strategies. Therefore, employing the One Health concept, it is recommended that veterinarians and medical personnel jointly address prevention and control strategies through enhanced surveillance, public sensitisation, and awareness raising.

## Funding

This research was funded in whole, or in part, by the 10.13039/100010269Wellcome Trust [Grant number 220211]. For the purpose of open access, the author has applied a CC BY public copyright license to any Author Accepted Manuscript version arising from this submission.

## Declaration of Competing Interest

The authors declare no conflict of interest.

## Data Availability

Data will be made available on request.
